# Metabolic changes in schizophrenia and human brain evolution

**DOI:** 10.1186/gb-2008-9-8-r124

**Published:** 2008-08-05

**Authors:** Philipp Khaitovich, Helen E Lockstone, Matthew T Wayland, Tsz M Tsang, Samantha D Jayatilaka, Arfu J Guo, Jie Zhou, Mehmet Somel, Laura W Harris, Elaine Holmes, Svante Pääbo, Sabine Bahn

**Affiliations:** 1 Partner Institute for Computational Biology, Shanghai Institutes for Biological Sciences, Chinese Academy of Sciences, Yue Yang Road, Shanghai, 200031, PR China; 2 Max-Planck-Institute for Evolutionary Anthropology, Deutscher Platz, D-04103 Leipzig, Germany; 3 Institute of Biotechnology, University of Cambridge, Tennis Court Road, Cambridge, CB2 1QT, UK; 4 Department of Biomolecular Medicine, Division of SORA, Imperial College London, SW7 2AZ, UK; 5 University of Science and Technology of China, Jinzhai Road, Hefei, 230026, PR China; 6 Shanghai Jiao Tong University, Dongchuan Road, Shanghai, 200240, PR China

## Abstract

**Background:**

Despite decades of research, the molecular changes responsible for the evolution of human cognitive abilities remain unknown. Comparative evolutionary studies provide detailed information about DNA sequence and mRNA expression differences between humans and other primates but, in the absence of other information, it has proved very difficult to identify molecular pathways relevant to human cognition.

**Results:**

Here, we compare changes in gene expression and metabolite concentrations in the human brain and compare them to the changes seen in a disorder known to affect human cognitive abilities, schizophrenia. We find that both genes and metabolites relating to energy metabolism and energy-expensive brain functions are altered in schizophrenia and, at the same time, appear to have changed rapidly during recent human evolution, probably as a result of positive selection.

**Conclusion:**

Our findings, along with several previous studies, suggest that the evolution of human cognitive abilities was accompanied by adaptive changes in brain metabolism, potentially pushing the human brain to the limit of its metabolic capabilities.

## Abstract 2

Human cognitive evolution involved genes implicated in energy metabolism and energy-expensive brain functions that are also altered in schizophrenia, suggesting that human brains may have reached their metabolic limit, with schizophrenia as a costly by-product.

## Background

During the last 5-7 million years of human evolution, the brain has changed dramatically, giving rise to our unique cognitive abilities. The molecular changes responsible for the evolution of these abilities remain unknown. Comparisons between humans and one of our closest living relatives, chimpanzees, conducted at the DNA sequence and gene expression levels have resulted in a vast catalogue of differences between the two species [1,2]. Still, as the overwhelming majority of these differences are likely to play no role in the evolution of human cognition, identification of the relevant differences is a daunting task [3-5].

Another factor impeding identification of the evolutionary changes related to human cognition is our insufficient knowledge of the molecular mechanisms underlying higher cognitive functions. This lack of knowledge is understandable, given the difficulty of studying human-specific cognitive functions in model organisms and, clearly, conducting functional experiments on humans is not possible. An alternative approach to the study of human brain function is through the investigation of naturally occurring dysfunctions. Apart from their direct health applications, studies of human cognitive dysfunctions represent a window into the molecular mechanisms underlying human brain function. Identification of such mechanisms using disease data, however, is complicated, as many observed changes are probably only indirectly associated with the affected functions.

In this study, we attempted to identify molecular mechanisms involved in the evolution of human-specific cognitive abilities by combining biological data from two research directions: evolutionary and medical. Firstly, we identify the molecular changes that took place on the human evolutionary lineage, presumably due to positive selection. Secondly, we consider molecular changes observed in schizophrenia, a psychiatric disorder believed to affect such human cognitive functions as the capacity for complex social relations and language [6-12]. Combining the two datasets, we test the following prediction: if a cognitive disorder, such as schizophrenia, affects recently evolved biological processes underlying human-specific cognitive abilities, we anticipate finding a significant overlap between the recent evolutionary and the pathological changes. Furthermore, if such significant overlap is observed, the overlapping biological processes may provide insights into molecular changes important for the evolution and maintenance of human-specific cognitive abilities.

## Results

**Figure 1:**
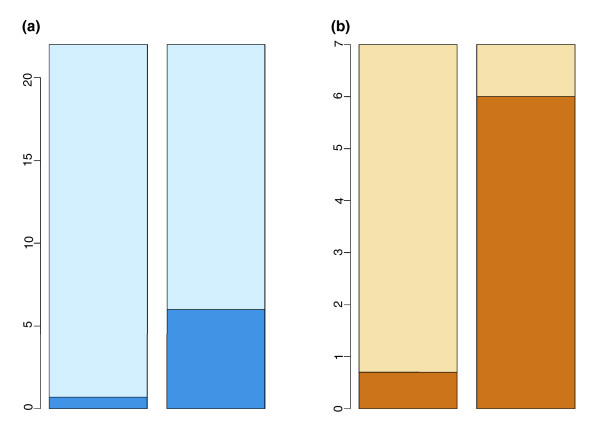
The proportion of biological processes showing evidence of recent positive selection on the human lineage that is differentially expressed in schizophrenia. The height of the bar represents the number of GO groups showing evidence of recent positive selection on the human lineage; **(a) **all 22 and **(b) **the 7 relating to energy metabolism. The darker shade of color represents the number of GO groups differentially expressed in schizophrenia among the 22 or the 7 GO groups (Wilcoxon rank sum test, *p *< 0.03, FDR = 11%). Left bar, expected by chance; right bar, observed.

In order to select human-specific evolutionary changes, we used the published list of 22 biological processes showing evidence of positive selection in terms of their mRNA expression levels in brain during recent human evolution [13]. Next, we tested whether expression of genes contained in these functional categories is altered in schizophrenia to a greater extent than expected by chance. To do this, we ranked 16,815 genes expressed in brain in order of probability of differential expression in schizophrenia, using data from a meta-analysis of 105 individuals profiled on 4 different microarray platforms in 6 independent studies [14]. We found that 6 of the 22 positively selected biological processes are significantly enriched in genes differentially expressed in schizophrenia (Wilcoxon rank sum test, *p *< 0.03, false discovery rate (FDR) = 11%), while only 0.7 would be expected to show such an enrichment by chance (Figure 1; Table S2 in Additional data file 1; Materials and methods). Strikingly, all six of these biological processes are related to energy metabolism. This is highly unexpected, given that there were only 7 biological processes containing genes involved in energy metabolism among the 22 positively selected categories (Figure 1; Table S2 in Additional data file 1). The mRNA expression changes observed in schizophrenia appear to be distributed approximately equally in respect to the direction of change, pointing towards a general dysregulation of these processes in the disease rather than a coordinated change (Table S3 in Additional data file 1).

**Figure 2:**
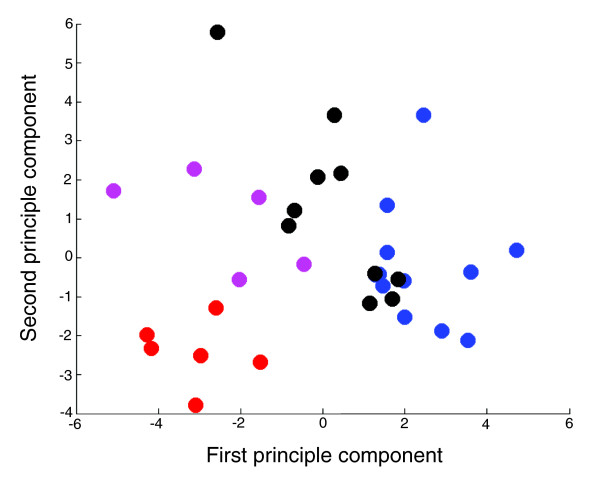
Principal component analysis of the metabolite abundance profiles in 33 individuals. The analysis is based on 21 detected metabolites. Each point represents an individual. The colors indicate: blue, human controls; black, human schizophrenia patients; purple, chimpanzees; red, rhesus macaques.

To investigate this further, we directly studied brain metabolism in prefrontal cortex of human schizophrenia patients (*N* = 10) and healthy controls (*N* = 12), as well as in two species of non-human primates, chimpanzees (*N* = 5) and rhesus macaques (*N* = 6), using ^1^H NMR spectroscopy (Materials and methods). This approach allowed the measurement of the relative concentrations of 21 distinct small metabolites/metabolite groups in all brain tissue samples studied, 20 of which could be unambiguously identified using public annotation (Table 1 and Materials and methods). Even with this relatively small number of metabolites, we clearly observe systematic differences in metabolite concentrations among the 4 sample groups (Figure 2), which account for more than 43% of total variation (by analysis of variance (ANOVA)). Neither differences in age or sex between species (Figure S1 in Additional data file 1), medication in schizophrenia patients nor differences in *post mortem *interval among samples accounted for these differences (Materials and methods). Thus, metabolite profiles of the brain appear biochemically distinct among such closely related primate species as humans, chimpanzees and rhesus macaques.

**Table 1:** Detected metabolites and metabolite groups

			Effect size^‡^		
			
Metabolite group	Number of peaks*	*t*-test *p*-value^†^	H_sch_/H_c_	H_c_/C	H_c_/R
Creatine	2	0.000	2.3	-2.3	-4.9
Lactate	6	0.005	1.5	-2.7	-0.6
Phosphocholine	1	0.034	1.0	-1.4	-0.3
Glycerophosphocholine	1	0.042	1.0	-1.5	-0.7
*N*-acetylaspartate	5	0.040	0.9	-2.2	-1.8
Acetate	1	0.025	-1.1	-0.3	0.1
Glycine	1	0.024	-1.1	3.3	4.2
Choline	1	0.010	-1.4	4.0	3.4
Unknown^§^	6	0.002	-1.5	2.8	6.2
Taurine	3	0.080			
Glutamate/glutamine 1^§¶^	4	0.114			
Glutamate/glutamine 2^¶^	4	0.130			
Glutamine^§^	4	0.280			
Glutamate 1^§¶^	3	0.381			
*Scyllo*-inositol	1	0.404			
Gamma-aminobutyric acid	5	0.470			
*Myo*-inositol	9	0.630			
Glutamate/proline	1	0.710			
*Myo*-inositol/taurine	3	0.797			
Glutamate 2^¶^	5	0.841			
*N*-acetylaspartylglutamate	1	0.845			

*Number of peaks in the NMR spectrum corresponding to the metabolite/metabolite group. ^†^Comparison between metabolite concentrations in 10 human schizophrenia patients and 12 human control individuals. ^‡^Effect size was calculated as the difference between means of metabolite concentrations between the groups normalized to the average standard deviation within the group. Positive values indicate higher concentration in group one, negative values higher concentration in group two. H_c_, human controls; H_sch_, human schizophrenia patients; C, chimpanzees; R, rhesus macaques. ^§^These peaks show a high degree of spectral overlap with other unidentified baseline peaks. ^¶^Glutamine/glutamate and glutamate peaks were separated into two independent groups based on the intensity correlation analysis (see Materials and methods).

Metabolic processes altered in disorders affecting human-specific cognitive function, such as schizophrenia, may be the same ones that underwent adaptive evolutionary change to support these abilities. When comparing metabolite concentrations between schizophrenia patients and control individuals, we detected significant differences between the two groups for 9 out of 21 metabolites (*t*-test *p *< 0.05, *FDR *= 11%; Table 1). Thus, even though our study is based on a limited number of metabolites, this result confirms that brain metabolism is substantially affected in schizophrenia. The altered metabolites play key roles in energy metabolism (creatine, lactate), neurotransmission (choline, glycine) and lipid/cell membrane metabolism (acetate, choline, phosphocholine, glycerophosphocholine) (Table 1). All three of these critical cellular processes have been implicated in schizophrenia, for example, through the use of *in vivo *magnetic resonance spectroscopy techniques [15-18].

**Figure 3:**
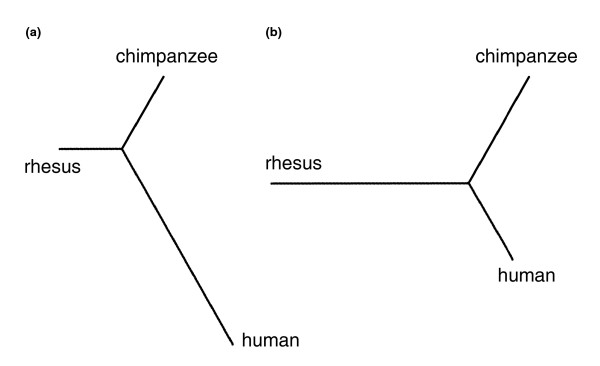
Divergence in metabolite abundance on the human and chimpanzee lineages. The trees are based on the abundance measurements of **(a) **9 metabolites with significant concentration difference between human controls and schizophrenia patients and **(b) **12 metabolites with no difference between these two groups. The trees were built using a neighbor-joining algorithm.

If schizophrenia affects biological process that also changed during human evolution, our hypothesis predicts that the 9 metabolites with significant concentration differences between schizophrenia patients and normal controls have evolved rapidly on the human lineage compared to the 12 metabolites not altered in the disease. In order to test this prediction, we measured changes in concentration on the human and chimpanzee lineages in the two metabolite groups using the rhesus macaque as an outgroup. In agreement with our prediction, we find that the ratio of the human to chimpanzee branch lengths in neighbor-joining tree reconstruction (Materials and methods) is more than three times greater for the 9-metabolite group than for the 12-metabolite group (2.8 versus 0.8; Figure 3). This difference is stable with respect to both metabolites and individuals used in the analysis, and is not due to the effect of any outliers (bootstrap analysis; Figure S2 in Additional data file 1; Materials and methods). Further, for eight out of the nine metabolites affected in schizophrenia, the direction of change in the disease is opposite to the direction of change in human evolution (*p *= 0.04, binomial test; Table 1).

Still, these observations are based on a rather small number of metabolites in *post mortem *brain samples and the bootstrap analysis cannot rule out an effect of an unknown systematic artifact. Thus, in order to test the result using independently generated data, we measured the extent of amino acid and mRNA expression divergence in genes involved in the biological processes related to the 9 metabolites significantly altered in schizophrenia and the 12 unaltered metabolites identified in our study. At the amino acid sequence level, we find genes contained in the Gene Ontology (GO) terms associated with the 9-metabolite group (*N* = 40) have significantly greater divergence between humans and chimpanzees than the genes associated with the 12-metabolite group (*N* = 81; *p *= 0.025, one-sided Wilcoxon test; Materials and methods). Similarly, comparing mRNA expression levels between brains of five humans and five chimpanzees (seven of these individuals were also investigated in the metabolite study), we find significantly higher expression divergence for genes associated with the 9-metabolite group than the genes associated with the 12-metabolite group (*p *= 0.05, one-sided Wilcoxon test; Materials and methods).

Greater amino acid or gene expression divergence can, however, indicate either positive selection or relaxation of selective constraint. In order to distinguish between these two possibilities, we used publicly available nucleotide polymorphism data to compare the extent of linkage disequilibrium (LD) - an indirect but unbiased measure of recent positive selection - between the two sets of genes [19]. LD reflects the extent of non-random association of alleles along chromosomes and positive selection is known to increase LD around the selected variant [20]. We indeed find that genes associated with the 9-metabolite group are associated with longer LD regions than the genes associated with the 12-metabolite group (*p *= 0.016, one-sided Wilcoxon test). Furthermore, this tendency can be observed in all three human populations tested: Africans, Chinese and Europeans (*p *= 0.076, 0.104 and 0.018, respectively; Figure S3 in Additional data file 1; Materials and methods). By contrast, we find no difference between the two groups of genes with respect to the local recombination rate - the main determinant of the LD extent in the absence of positive selection (*p *= 0.548, one-sided Wilcoxon test; Figure S3 in Additional data file 1). Thus, genes associated with metabolites that are altered in schizophrenia and fast evolving on the human lineage display greater amino acid sequence and expression divergence between humans and chimpanzees that may be due to recent positive selection in humans.

## Discussion

The aim of the present study was to explore the overlap between molecular changes observed in a disorder affecting human cognitive abilities and evolutionary changes observed on the human lineage in order to gain novel insights into the functional mechanisms underlying human cognition. We indeed find such an overlap at the mRNA expression level, and the vast majority of over-lapping changes relate to energy metabolism. We then measured metabolite concentrations in *post mortem *brain tissue from healthy human controls, human schizophrenia patients, chimpanzees and rhesus macaques. Again, we find that metabolic processes altered in the schizophrenia brain evolved rapidly on the human, but not on the chimpanzee, evolutionary lineage. In contrast, we find no such difference between the two lineages for the metabolic processes not affected by the disease. Further, we found that genes associated with fast evolving metabolic processes also show greater divergence between humans and chimpanzees at both the amino acid sequence and mRNA expression levels than the genes associated with metabolites not altered in schizophrenia. Both an excess of adaptive changes and a relaxation of selective constraint could cause such an increase in evolutionary divergence. However, the fact that we find signatures of recent positive selection in the vicinity of genes associated with fast evolving metabolic processes indicates that adaptive changes is the more parsimonious explanation.

Still, alternative explanations for these results need to be considered. It is possible that pathways relating to energy metabolism are altered in schizophrenia and evolutionary studies simply because mRNAs associated with these biological processes are more likely to be influenced by *post mortem *effects. This logic could also be applied to the metabolite study. Several arguments, however, refute this explanation.

First, in the metabolite study, schizophrenia and control samples were matched for age, brain pH, *post mortem *interval (Student's *t*-test, *p *= 0.31, *p *= 0.55, *p *= 0.15, respectively) and sex (Fisher's exact test, *p *= 0.65) (Table S1 in Additional data file 1). However, we cannot exclude the effect of antipsychotic medication on the observed metabolic differences in schizophrenia, even though the patient cohort chosen received relatively little medication (Table S1 in Additional data file 1). Still, both schizophrenia and the medications used to treat it are expected to target functional processes relevant to human-specific cognitive abilities.

Second, our main finding - rapid evolution of schizophrenia-affected metabolic processes on the human lineage - is based on a comparison of evolutionary rates for two metabolite groups measured within the same experiment. Thus, if this result were due to a confounding factor, such an artifact has to be specific to the particular biological processes, occur in the control but not in schizophrenia samples, or affect both mRNA and metabolite expression levels. Further, as there are no significant sampling differences between schizophrenia patients and normal controls with regard to parameters such as age, sex, *post mortem *interval or brain pH, the artifact has to be caused by an unknown sampling bias.

Third, we find greater amino acid divergence and an increased association with genomic signatures of recent positive selection in these biological processes. Even if *post mortem *effects or other technical artifacts can cause differences in mRNA and metabolite expression, they are unlikely to explain differences at the DNA or amino acid sequence levels.

Taken together, our results indicate that energy metabolism may play an important role in sustaining the cognitive functions specific to the human brain. This is not inconceivable, given that humans allocate around 20% of their total energy to the brain, compared to approximately 13% for non-human primates and 2-8% for other vertebrates [21]. An important role for metabolic changes in the establishment of human brain functionality is further implied by recent observations that genes related to neuronal function and energy metabolism show increased expression levels in humans compared to other primates [22,23]. Further, there are indications of positive selection for genes involved in energy metabolism in anthropoid primates and humans, in terms of amino acid composition [24] and elevated expression levels in brain [13], respectively. Recently, positive selection during human evolution was also shown to target the promoters of genes involved in glucose metabolism - the main source of energy for the brain [25].

At the same time, there is growing evidence that brain energy metabolism is altered in neuropsychiatric disorders, such as schizophrenia, in which human-specific cognitive abilities are impaired. Deficits in blood flow in the prefrontal cortex are consistently reported in schizophrenia patients relative to controls, particularly when performing complex cognitive tasks [26,27]. Furthermore, the altered metabolic activity correlates with the severity of negative symptoms and cognitive deficits [28]. Concordantly, several studies have identified down-regulation of numerous genes involved in energy metabolism in the schizophrenia *post mortem *brain [29-32].

Combining the two research fields, we find further indications supporting the crucial role of energy metabolism in the evolution and maintenance of human-specific cognitive abilities. The metabolites that changed their concentrations in brain during human evolution are involved in the most energy demanding processes of the human brain - maintenance of the membrane potential and the continual synthesis of neurotransmitters [33,34]. In human evolution, the disproportional increase in brain size would result in an increase in both the length and diameter of neuronal connections [35] and the number of synapses, further elevating energy demands associated with membrane potential maintenance and neurotransmitter turnover. Given that the relatively short time of about 2 million years since the increase in human brain size does not allow for much optimization, it is conceivable that the human brain is running very close to the limit of its metabolic capacities. As a consequence, any perturbation of normal energy metabolism levels may be expected to adversely affect brain function, leading to human cognitive dysfunctions. It would seem reasonable to suppose that energetically expensive neurons would be most susceptible to such changes. Supporting this notion, schizophrenia is associated with structural and functional deficits in the fronto-temporal and fronto-parietal circuits [11], which are connected by long-range projection neurons displaying high-energy characteristics such as long, highly myelinated axons and fast firing rates [34].

We must note, however, that both schizophrenia and evolutionary studies conducted so far, including the study presented here, provide no direct link between metabolic changes, such as changes in energy metabolism, and cognitive phenotype. This limitation is inherent to all studies of human-specific phenotypic features that cannot be approached experimentally. In addition, cognitive changes observed in schizophrenia do not affect the full spectrum of human-specific cognitive traits and certainly do not recapitulate the extent of differences between humans and other primates. Thus, studies involving other human cognitive disorders are necessary in order to clarify the relationship between metabolic changes and human cognitive features.

Further, our results do not allow us to distinguish whether the positive selection on metabolic processes has acted on the molecular changes underlying increased cognitive abilities of the human brain or reflects the need for optimizing brain metabolic activity following an increase in brain size. As the signatures of positive selection we can detect are restricted to the last 200,000 years [36], almost 2 million years after the increase in human brain size, the former explanation may be more plausible. On the other hand, it is conceivable that optimization of the human brain metabolic activity following an increase in size is still ongoing.

Lastly, the small number of metabolites identified in this study also precludes us from distinguishing evolutionary changes directly related to energy metabolism and the changes affecting other aspects of brain functionality, such as signal transduction or neurotransmitter turnover. Still, the fact that potential human-specific adaptations can already be seen among 21 metabolites studied here indicates that many more metabolic changes are likely to be associated with the rapid brain size increase during human evolution. Thus, further work involving greater numbers of samples and metabolites, and the study of other neuropsychiatric disorders is certainly necessary.

## Conclusion

In this study we find a disproportionately large overlap between processes that have changed during human evolution and biological processes affected in schizophrenia. Genes relating to energy metabolism are particularly implicated for both the evolution and maintenance of human-specific cognitive abilities.

Using ^1^H NMR spectroscopy, we find evidence that metabolites significantly altered in schizophrenia have changed more on the human lineage than those that are unaltered. Furthermore, genes related to the significantly altered metabolites show greater sequence and mRNA expression divergence between humans and chimpanzees, as well as indications of positive selection in humans, compared to genes related to the unaltered metabolites.

Taken together, these findings indicate that changes in human brain metabolism may have been an important step in the evolution of human cognitive abilities. Our results are consistent with the theory that schizophrenia is a costly by-product of human brain evolution [11,37].

## Materials and methods

### Samples

All samples used in this study were taken from the middle third of the middle frontal gyrus and the most rostral portion of the inferior frontal gyrus of the human prefrontal cortex approximately corresponding to Brodmann area 46, and from the equivalent region in the non-human primates. Human *post mortem *brain tissue samples from ten schizophrenia patients and ten normal controls were obtained from the Stanley Medical Research Institute (Bethesda, USA), comprising a subset of the Array Collection that was well-matched for demographic variables (Table S1 in Additional data file 1). All schizophrenia patients had been treated to some extent with antipsychotic medication (typically with two or three different antipsychotics). However, efforts were made to include individuals that had received relatively little treatment overall, as measured by fluphenazine milligram equivalents. In addition, two normal control brain samples were obtained from the National Disease Research Interchange (Philadelphia, USA). Informed consent for use of the human tissues for research was obtained in writing from all donors or the next of kin. Chimpanzee samples (*N* = 5) were obtained from the Yerkes Primate Center (Atlanta, USA) and from the Biomedical Primate Research Centre (Rijswijk, Netherlands). All chimpanzee individuals used in this study belonged to the Eastern chimpanzee population. Rhesus macaque samples (*N* = 6) were obtained from the German Primate Center (Goettingen, Germany). All non-human primates used in this study suffered sudden deaths for reasons other than their participation in this study and without any relation to the tissues used. No samples showed any detectable RNA degradation, as measured using an Agilent Bioanalyzer (Agilent Technologies, Palo Alto, CA, USA), indicating good tissue preservation. Details of all samples, including age, sex, brain pH and *post mortem *interval are given in Table S1 in Additional data file 1.

### Gene expression data analysis

Data were obtained from the Stanley Medical Research Institute's online genomics database [14], which represents the most comprehensive repository of transcriptomics data for neuropsychiatric disorders, including schizophrenia. This database was derived from two sets of brain samples: the Stanley Array collection and the Stanley Consortium collection. For this study data were selected from the Stanley Array collection only, since the tissue homogenate samples in this set were taken from the same brain region (prefrontal cortex, brain region corresponding to Brodmann area 46) that was analyzed in the comparative transcriptomics study [13]. The Stanley Array collection comprises samples from a population of 105 individuals, profiled on 4 different microarray platforms, in 6 independent studies. This dataset has been summarized in a meta-analysis in which the effects of confounding demographic variables (for example, age, *post mortem *interval, tissue pH, and so on) were controlled using a linear regression method [14]. For each of the 16,815 genes (as defined by EntrezGene), the meta-analysis yielded a probability of differential expression in schizophrenia.

The aim of our analysis was to determine whether or not the 22 GO groups showing evidence of recent positive selection on the human lineage previously identified [13] are differentially expressed in schizophrenia. All of the assayed genes were ranked in order of increasing *p*-value for the probability of differential expression and any GO category containing more highly ranked genes than would be expected by chance was considered to be differentially expressed. Specifically, for each of the 22 GO categories showing evidence of positive selection, the ranks of the genes in the GO category were compared to the ranks of all other assayed genes using a one-sided Wilcoxon Rank Sum test. The false discovery rate was calculated by randomly permuting gene rank assignments 10,000 times. This permutation analysis also provided an estimate of the probability of finding an equal or greater number of differentially expressed GO categories than was observed in the real data. Full details of the results of this analysis are given in Table S2 in Additional data file 1.

### NMR spectroscopic analysis

#### Preparation of tissue extracts from brain samples

For each individual used in this study, approximately 60-80 mg prefrontal cortex tissue (Brodmann area 46) was dissected from a frozen brain sample on dry ice without thawing. Special care was taken to avoid differences in the gray matter to white matter ratio between samples and processed randomly with respect to species or disease. Aqueous components were extracted from brain tissue samples using previously described techniques [38,39]. Frozen tissue samples were individually homogenized in 1 ml of acetonitrile/de-ionized water mix (1:1) and then centrifuged at 4,800 g for 10 minutes. The supernatants were transferred to separate eppendorf tubes to allow full evaporation of the acetonitrile over 24 h before being lyophilized. For ^1^H NMR spectroscopic analysis, samples were reconstituted in 600 μl deuterated water (95% D_2_O:5% H_2_O).

#### ^1^H NMR spectroscopic acquisition of aqueous brain extracts

Supernatant (600 μl) was placed in a 5 mm outer diameter NMR tube. ^1^H NMR spectra were acquired on each sample at 600.13 MHz on a Bruker AMV600 spectrometer (Rheinstetten, Germany), equipped with a TXI (triple channel inverse) probe, at ambient probe temperature (300 K). A standard one-dimensional (1D) pulse sequence was used (recycle delay-90°-*t*_1_-90°-*t*_m_-90°-acquire free induction decay). The water signal was suppressed by irradiation during a recycle delay of 2 s, and mixing time (*t*_m_) of 150 ms. *t*_1 _was set to 3 μs and the 90° pulse length was adjusted to approximately 10 μs. For each sample, 64 transients were accumulated into 32K data point using a spectral width of 20 ppm. Prior to Fourier transformation, all free induction decays were multiplied by an exponential function equivalent to a line broadening of 0.3 Hz.

#### Data processing

Using an in-house developed MATLAB [40] routine, NMR spectra were digitized into 29,999 data points over the range of δ 0.5-9.0 excluding the water region (δ 4.5-6.4) (Table S4 in Additional data file 1). The resulting NMR spectra were normalized to the same average intensity. Because aqueous brain extracts were used for the NMR measurements, only the part of the spectrum containing signals of soluble metabolites was analyzed in the subsequent steps. This resulted in the reduction of each spectrum to 16,000 data points. Next, in order to prevent measurement artifacts caused by slight shifts in the metabolite peak positions among spectra, peaks in all 33 spectra were aligned with the 'beam search' algorithm with default parameters [41,42], using one randomly chosen typical individual spectrum as a standard.

Following peak alignment, the area under each peak was calculated using the 'interp1' function from the MATLAB software package. In this function, a curve is first fitted to the peak outline and the area is integrated by dividing it into small rectangles. Fitting the curve first allows better area integration by dividing it into smaller intervals. In our calculation, we allowed five times greater data point density in the peak area than contained in the original spectra. In the area integration step, the problem of calculating areas of overlapping peaks is encountered. This was resolved by fitting a line to the linear part of the slope of the overlapping peak in order to extrapolate the peak shape in the region of overlap. In each case, the line fitting was performed using ten or more data points to ensure reliable extrapolation.

Next, metabolite peaks detectable above the background in all 33 spectra were determined. The background value was calculated as an average intensity of the hydrophobic area of the NMR spectra. Following this approach, 67 distinct peaks could be identified, including virtually all peaks discernable in the spectra as confirmed by the manual data inspection. Still, this approach excluded metabolite peaks not present in all species or all groups of individuals, making our estimates of between group differences more conservative. Most notably, this approach excluded a strong peak of unknown metabolite detectable in rhesus macaques, but not in the other species analyzed. The resulting peak areas were base-two-logarithm transformed and the sum of all peak areas for each individual was scaled to one.

After peak detection, the 67 peaks were assigned to their metabolites using published annotation [43-45]. Following this procedure, 61 peaks could be assigned to 20 metabolites or metabolite groups. Of these, twelve were represented by more than one peak. Since peaks corresponding to the same metabolite are expected to change concordantly among the 33 spectra analyzed, we calculated the correlation between all peak pairs to confirm the existing annotation and to group the remaining 6 peaks. For all but two metabolite groups, (glutamate/glutamine and glutamate), the abundance measurements from all peaks assigned to the same metabolite correlated significantly with one another (*p *< 0.05, Spearman correlation test) in agreement with the existing annotation. Peaks assigned to glutamate/glutamine and glutamate separated into two groups, likely due to the high degree of spectral overlap with resonances of other compounds observed for these peaks. Because the influence of other compounds resulted in two clearly distinctive patterns of intensity change among the samples (*p *< 0.05, Spearman correlation test), we considered them as two independent metabolic groups in the subsequent analysis.

Further, the six unannotated peaks were all significantly correlated with each other (*p *< 0.05, Spearman correlation test) and were thus grouped as one additional metabolite group. The positions of the unannotated peaks fall within the spectral region that has been previously assigned to *myo*-inositol. In our analysis, however, the six unannotated peaks and the nine peaks that can be confidently assigned to *myo*-inositol form two distinct correlation patterns based on the peak intensity changes among samples and show very different behavior in terms of differences between schizophrenia patients and the normal control group. Thus, these were also considered as two independent metabolic groups in the subsequent analysis. Full details of spectra peak positions and metabolite assignments are given in Table S5 in Additional data file 1.

### Principal components analysis of metabonomic data

Principal components analysis (PCA) was performed using the MATLAB software package. All metabolite peaks were scaled to mean equal zero and standard deviation equal one among all samples to ensure the same contribution to the separation for all peaks. Intensities of peaks corresponding to the same metabolite were averaged prior to PCA. The PCA result was the same using individual peak data (data not shown). The influence of age, medication, *post mortem *interval and sex on the species separation was tested by redrawing PCA plots using data residuals after linear regression analysis with age, amount of medication or *post mortem *interval as a continuous variable or after ANOVA with factor 'sex'. The exact *post mortem *interval for non-human primate samples was not known precisely; a value of two hours was used for all non-human samples in this analysis, based on the average time taken for the autopsy procedure. None of these factors were found to affect the distinct species separation. The proportion of total variation explained by the species and the disease was estimated using data residuals after ANOVA with four sample groups as a factor.

### Disease analysis of metabonomic data

Metabolite concentrations in the human control subjects (*N* = 12) and schizophrenia patients (*N* = 10) were compared using Student's *t*-test on scaled intensities of 21 metabolites. The FDR was determined by randomly permuting individual assignments to the two tested groups 5,000 times. At the nominal *t*-test *p*-value of 0.05, the FDR equaled 10.8%.

### Phylogenetic analysis of metabonomic data

The trees were built and drawn with the PHYLIP software package [46] using a neighbor-joining algorithm and based on the pairwise Euclidian distances between average metabolite abundance measurements in each species. Prior to the distance calculation, all metabolite peaks were scaled to mean equal zero and standard deviation equal one among all samples to ensure the same contribution to the separation for all peaks.

### Genome and mRNA data analysis

To further test the finding that metabolites significantly altered in schizophrenia patients compared to controls have changed more on the human lineage than unaltered metabolites, the corresponding genes were also investigated. The assignment of genes to metabolites was performed using biological process annotation provided by the GO consortium [47]. First, the GO terms associated with each metabolite were identified using a key word search in the GO database [48]. The following keywords were used for the significantly altered metabolites in schizophrenia: 'choline', 'creatine', 'acetate', 'glycerophosphocholine', 'lactate', 'glycine', 'NAA', '*N*-acetyl-aspartate', 'phosphocholine'. The keywords for unaltered metabolites in schizophrenia were: 'gamma-aminobutyric acid', 'glutamate', 'glutamine', 'proline', '*myo*-inositol', 'taurine', '*scyllo*-inositol'. Then, genes associated with these GO terms were identified using Ensembl Biomart [49]. This resulted in the identification of 48 genes associated with the 9 metabolites that significantly differed in concentration in schizophrenia patients compared to normal controls (group 1) and 96 genes associated with the remaining 12 (group 2) (Table S6 in Additional data file 1).

In order to test whether genes associated with the two metabolite groups differ in their mRNA expression divergence between humans and chimpanzees, we measured gene expression profiles in five human and five chimpanzee samples derived from the same brain region as used for the metabolite concentration measurements (corresponding to the Brodmann area 46) using Affymetrix Human Exon arrays. All chimpanzee individuals and two out of five human individuals were shared between the mRNA and metabolite measurements. Prior to analysis, all array probes that did not match both the chimpanzee and the human genomes were masked and the microarray intensity signals were normalized and processed as described elsewhere [2]. The resulting expression intensities for genes associated with group 1 and group 2 metabolites are listed in Tables S7 and S8 in Additional data file 1.

Positive selection acting on protein sequence evolution may be recognized from measurements of amino acid divergence, such as K_a_/K_i_, and from signatures of nucleotide polymorphism reflecting non-neutral evolution, such as extent of LD. Amino acid divergence tables (K_a_/K_i_) were obtained from The Chimpanzee Sequencing and Analysis Consortium [50]. Of the 13,454 genes contained in this dataset, 40 genes are associated with the group 1 metabolites, and 81 genes with the group 2 metabolites. The discrepancy with the total number of genes identified by the keyword search of the GO database described above is due to differential data availability from the different public sources. LD and recombination rate tables were downloaded from Perlegen [51] and UCSC Human Genome Browser [52], respectively. The recombination rate data represents calculated sex-averaged rate values based on the deCODE genetic map obtained using microsatellite markers mapping [53]. LD and recombination rate measurements for each gene were calculated as described elsewhere [13] with no modifications. Both LD and recombination group measurements were available for the 40 genes associated with group 1 metabolites and for the 81 genes associated with group 2 metabolites.

## Abbreviations

ANOVA, analysis of variance; FDR, false discovery rate; GO, Gene Ontology; LD, linkage disequilibrium; NAA, N-acetyl-aspartate; PCA, principal components analysis.

## Authors' contributions

TMT and SDJ carried out the ^1^H NMR experiments. PK, MTW, TMT, SDJ, AJG, JZ, and MS carried out data analysis. PK, EH, SP, and SB conceived of the study, and participated in its design and coordination. PK, HEL, MTW, TMT, LWH, EH, SP, and SB wrote the manuscript. All authors read and approved the final manuscript.

## Additional data files

The following additional data are available with this paper. Additional data file 1 contains all supplementary figures and tables. Figure S1 shows the PCA of the metabolite abundance profile residuals in 33 individuals after sex and age linear regression. Figure S2 shows the bootstrap analysis of the ratio of human/chimpanzee lineage length. Figure S3 shows the extent of the LD and the recombination rate for genes associated with metabolites affected and not affected in schizophrenia. Table S1 lists sample information. Table S2 is a representation of schizophrenia-related expression changes in GO categories positively selected during human evolution. Table S3 lists GO groups showing excess of expression changes in both schizophrenia and human evolution. Table S4 provides ^1^H NMR spectra for 33 samples. Table S5 list the assignments of NMR spectra peaks to metabolites and metabolite groups. Table S6 lists genes associated with fast-evolving and slow-evolving metabolite groups. Table S7 lists mRNA expression of genes associated with metabolites significantly altered in schizophrenia. Table S8 lists mRNA expression of genes associated with metabolites not altered in schizophrenia.

## Supplementary Material

Additional data file 1 Figure S1 shows the PCA of the metabolite abundance profile residuals in 33 individuals after sex and age linear regression. Figure S2 shows the bootstrap analysis of the ratio of human/chimpanzee lineage length. Figure S3 shows the extent of the LD and the recombination rate for genes associated with metabolites affected and not affected in schizophrenia. Table S1 lists sample information. Table S2 is a representation of schizophrenia-related expression changes in GO categories positively selected during human evolution. Table S3 lists GO groups showing excess of expression changes in both schizophrenia and human evolution. Table S4 provides ^1^H NMR spectra for 33 samples. Table S5 list the assignments of NMR spectra peaks to metabolites and metabolite groups. Table S6 lists genes associated with fast-evolving and slow-evolving metabolite groups. Table S7 lists mRNA expression of genes associated with metabolites significantly altered in schizophrenia. Table S8 lists mRNA expression of genes associated with metabolites not altered in schizophrenia. Click here for file
