# Supplementary material for: Metabolic changes in schizophrenia and human brain evolution
Source: Genome Biol. 2008 Aug 5;9(8):R124. doi: 10.1186/gb-2008-9-8-r124 (PMC2575514; doi:10.1186/gb-2008-9-8-r124)
Supplement: Additional data file 1 — Figure S1 shows the PCA of the metabolite abundance profile residuals in 33 individuals after sex and age linear regression. Figure S2 shows the bootstrap analysis of the ratio of human/chimpanzee lineage length. Figure S3 shows the extent of the LD and the recombination rate for genes associated with metabolites affected and not affected in schizophrenia. Table S1 lists sample information. Table S2 is a representation of schizophrenia-related expression changes in GO categories positively selected during human evolution. Table S3 lists GO groups showing excess of expression changes in both schizophrenia and human evolution. Table S4 provides 1H NMR spectra for 33 samples. Table S5 list the assignments of NMR spectra peaks to metabolites and metabolite groups. Table S6 lists genes associated with fast-evolving and slow-evolving metabolite groups. Table S7 lists mRNA expression of genes associated with metabolites significantly altered in schizophrenia. Table S8 lists mRNA expression of genes associated with metabolites not altered in schizophrenia. [file gb-2008-9-8-r124-S1.doc]

**Supporting Information**

**Metabolic changes in schizophrenia and human brain evolution**

Philipp Khaitovich, Helen E. Lockstone, Matthew T. Wayland, Tsz Mon Tsang, Samantha D Jayatilaka, Arfu J. Guo, Jie Zhou, Mehmet Somel, Laura W Harris, Elaine Holmes, Svante Pääbo and Sabine Bahn

**I. Supplementary Figures**

**
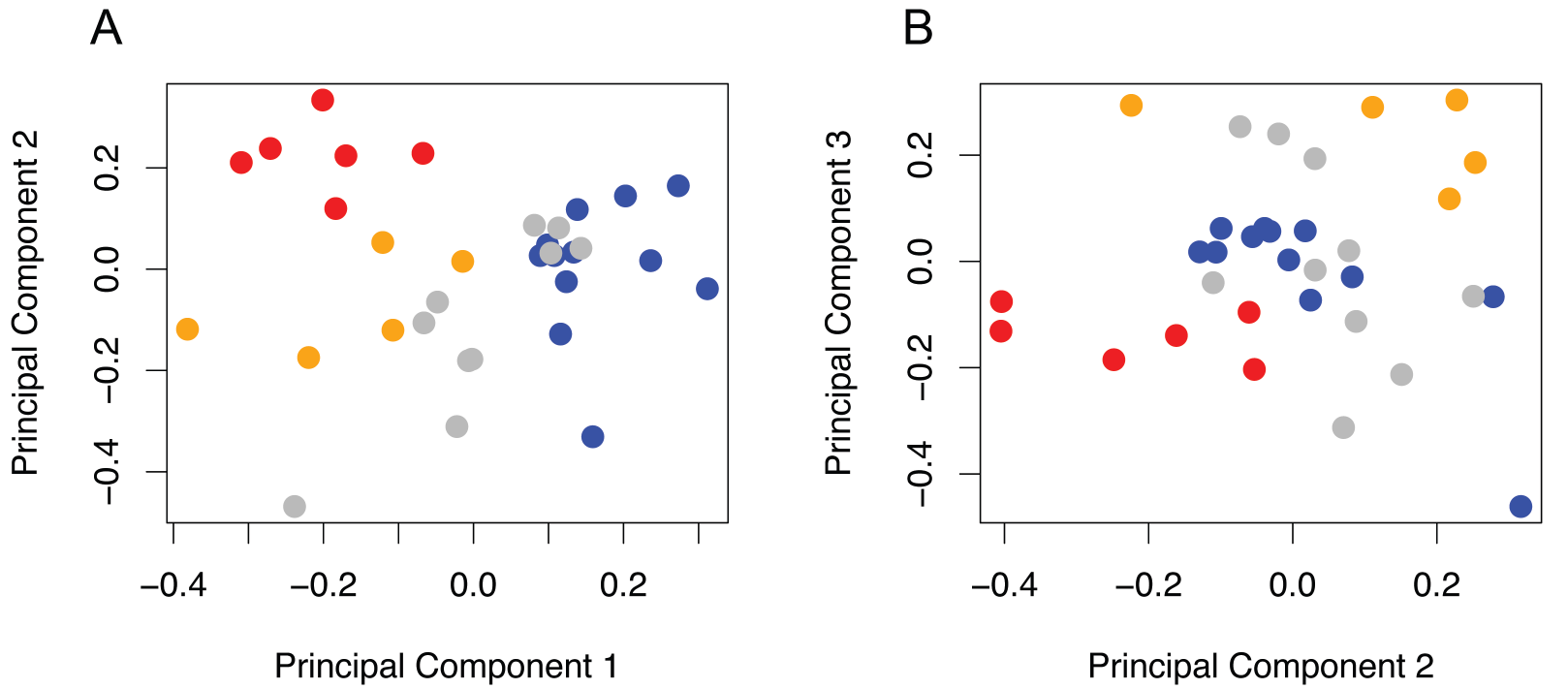
**

**Figure S1.** **Principal component analysis of the metabolite abundance profiles residuals in 33 individuals after sex and age linear regression analysis**

Metabolite abundance residuals calculation for sex (**A**) and age (**B**) effects (Materials and methods). The analysis is based on 21 detected metabolites. Each point represents an individual. The colors indicate: blue – human controls, gray – human schizophrenia patients, orange – chimpanzees, red – rhesus macaques.


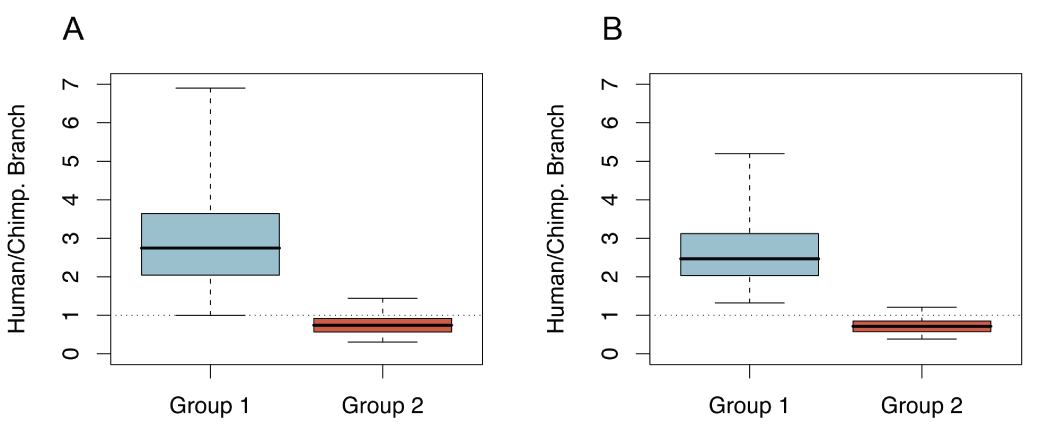


**Figure S2. Bootstrap analysis of the ratio of human/chimpanzee lineage length**

The length of the lineages was estimated using neighbor-joining algorithm. Bootstrap analysis was performed 500 times over metabolites (**A**) or over individuals within each species (**B**). The bootstrap results are shown for the 9 metabolites with significant concentration difference between human controls and schizophrenia patients (Group 1, blue) and 12 metabolites with no difference between these two groups (Group 2, red). The box area represents the range between the data first and the third quartiles. The whiskers indicate the borders of the 95% confidence interval.

**
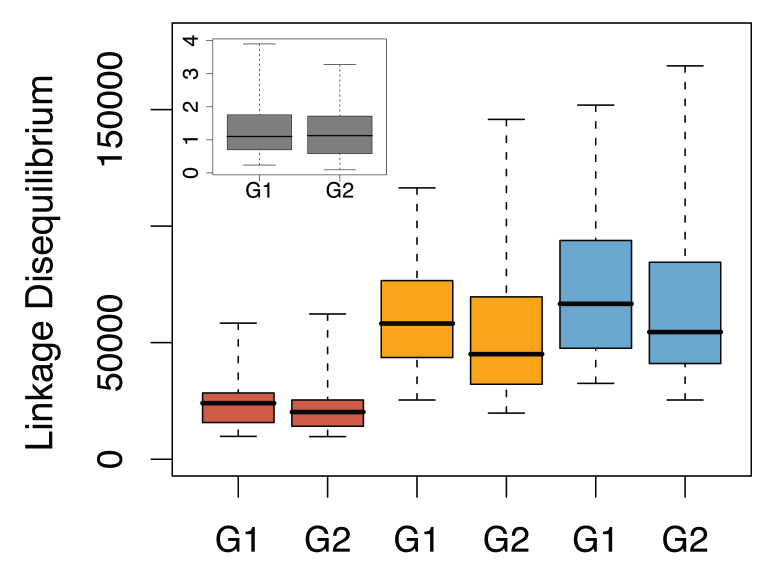
**

**Figure S3. Extent of the linkage disequilibrium and the recombination rate for genes associated with metabolites affected and not affected in schizophrenia**

The main plot shows distribution of the linkage disequilibrium for 40 genes associated with 9 metabolites affected in schizophrenia (**G1**) and 81 genes associated with 12 metabolites not affected in schizophrenia (**G2**) in three human populations: Africans (red), Europeans (yellow), and Chinese (blue). The insert shows distribution of the recombination rate measurements for the same genes. The thick line inside the box shows median value, the box covers data distribution between the first and the third quartiles, and the whiskers indicate 95% of the values distribution.

**II. Supplementary Tables**

**Table S3.** GO groups showing excess of expression changes in both schizophrenia and human evolution

|  | GO id | Genes* | Up** | Down | p-value† | adjusted p-value‡ | GO name |
| --- | --- | --- | --- | --- | --- | --- | --- |
|  | | GO:0008152 | | --- | | 591 | 275 | 316 | 0.0998 | 0.1894 | metabolism |
|  | | GO:0006631 | | --- | | 15 | 11 | 4 | 0.1185 | 0.1894 | fatty acid metabolism |
|  | | GO:0019752 | | --- | | 51 | 25 | 26 | 1.0000 | 1.0000 | carboxylic acid metabolism |
|  | | GO:0006082 | | --- | | 53 | 27 | 26 | 1.0000 | 1.0000 | organic acid metabolism |
|  | | GO:0006091 | | --- | | 43 | 16 | 27 | 0.1263 | 0.1894 | generation of precursor metabolites and energy |
|  | | GO:0015980 | | --- | | 16 | 4 | 12 | 0.0768 | 0.1894 | energy derivation by oxidation of organic compounds |
|  |  |  |  |  |  |  |  |
|  | metabolism |  |  |  | *Number of differentially expressed genes in a given GO category (t-test at an FDR of 25%). | | |
|  | fatty and carboxylic acid metabolism | | |  | ** Up- and Down- regulation in schizophrenia patients compared to controls. | | |
|  | electron transport and energy pathways | | |  | †p-value from Binomial test for overrepresentation of up- and down-regulated genes in a given GO category. | | |
|  |  |  |  |  | ‡FDR adjusted p-value from Binomial test for overrepresentation of up- and down-regulated genes in a given GO category. | | |
|  | |  | | --- | |  |  |  |

**Table S4** could not be included in this document due to size and can be downloaded from http://www.picb.ac.cn/Comparative/metabolites001/tableS3.zip

| **Table S7.** mRNA expression of genes associated with metabolites significantly altered in schizophrenia | | | | | | | | | | |  |
| --- | --- | --- | --- | --- | --- | --- | --- | --- | --- | --- | --- |
|  |  |  |  |  |  |  |  |  |  |  |  |
| Ensembl Gene ID | HGNC Symbol | Chimpanzee | | | | | Human | | | | |
|  |  | C1 | C2 | C3 | C4 | C5 | H1 | H2 | H3 | H4 | H5 |
| ENSG00000115665 | SLC5A7 | 2.03 | 2.09 | 1.94 | 1.77 | 2.12 | 2.12 | 1.91 | 1.96 | 1.94 | 2.14 |
| ENSG00000114353 | GNAI2 | 2.18 | 2.29 | 2.21 | 2.22 | 2.00 | 2.31 | 2.39 | 2.37 | 2.30 | 2.27 |
| ENSG00000206561 | COLQ | 2.01 | 2.15 | 2.25 | 2.23 | 2.20 | 2.12 | 2.19 | 2.18 | 2.10 | 2.21 |
| ENSG00000188985 | DHFR | 1.52 | 2.07 | 2.16 | 2.13 | 1.80 | 2.12 | 2.45 | 2.22 | 2.02 | 2.36 |
| ENSG00000161217 | PCYT1A | 2.17 | 2.27 | 2.14 | 2.29 | 2.16 | 2.37 | 2.39 | 2.34 | 2.36 | 2.32 |
| ENSG00000109738 | GLRB | 2.56 | 2.55 | 2.37 | 2.37 | 2.59 | 2.49 | 2.23 | 2.43 | 2.39 | 2.40 |
| ENSG00000164303 | ENPP6 | 2.09 | 2.24 | 1.99 | 2.08 | 1.67 | 2.07 | 2.19 | 1.99 | 2.05 | 1.91 |
| ENSG00000132837 | DMGDH | 1.96 | 1.88 | 1.14 | 1.85 | 0.76 | 1.90 | 1.29 | 1.99 | 2.07 | 2.03 |
| ENSG00000087085 | ACHE | 2.41 | 2.27 | 2.13 | 2.26 | 2.01 | 2.22 | 2.22 | 2.28 | 2.19 | 2.12 |
| ENSG00000147432 | CHRNB3 | 1.91 | 1.97 | 1.57 | 1.90 | 2.00 | 2.03 | 1.96 | 2.07 | 1.76 | 1.90 |
| ENSG00000107165 | TYRP1 | 1.57 | 1.92 | 0.98 | 1.90 | 1.87 | 2.01 | 1.66 | 1.86 | 2.01 | 2.23 |
| ENSG00000070214 | SLC44A1 | 2.89 | 2.85 | 2.83 | 2.85 | 2.67 | 2.63 | 3.09 | 2.79 | 2.87 | 2.72 |
| ENSG00000178445 | GLDC | 2.20 | 2.18 | 2.39 | 2.26 | 2.11 | 2.14 | 2.17 | 2.33 | 2.24 | 2.19 |
| ENSG00000136881 | BAAT | 2.15 | 2.06 | 2.15 | 2.09 | 1.97 | 1.02 | 0.79 | 1.62 | 1.35 | 1.26 |
| ENSG00000123453 | SARDH | 1.71 | 1.71 | 1.62 | 1.64 | 1.57 | 1.56 | 1.48 | 1.59 | 1.24 | 1.62 |
| ENSG00000166275 | C10orf32 | 1.09 | 1.65 | 1.13 | 1.10 | 1.62 | 2.09 | 2.21 | 2.08 | 1.98 | 2.17 |
| ENSG00000166275 | AS3MT | 1.09 | 1.65 | 1.13 | 1.10 | 1.62 | 2.09 | 2.21 | 2.08 | 1.98 | 2.17 |
| ENSG00000166275 | C10orf32 | 1.96 | 1.92 | 1.69 | 1.83 | 2.01 | 2.32 | 2.16 | 2.01 | 2.10 | 1.99 |
| ENSG00000166275 | AS3MT | 1.96 | 1.92 | 1.69 | 1.83 | 2.01 | 2.32 | 2.16 | 2.01 | 2.10 | 1.99 |
| ENSG00000168539 | CHRM1 | 2.36 | 2.34 | 2.42 | 2.46 | 2.44 | 2.48 | 2.28 | 2.26 | 2.39 | 2.34 |
| ENSG00000110721 | CHKA | 2.34 | 2.23 | 2.32 | 2.31 | 2.31 | 2.28 | 2.41 | 2.33 | 2.36 | 2.37 |
| ENSG00000182199 | SHMT2 | 2.32 | 2.55 | 2.30 | 2.34 | 2.27 | 2.21 | 2.15 | 2.26 | 2.23 | 2.19 |
| ENSG00000111666 | CHPT1 | 2.40 | 2.42 | 2.32 | 2.28 | 2.45 | 2.33 | 2.41 | 2.41 | 2.42 | 2.51 |
| ENSG00000081760 | AACS | 2.42 | 2.29 | 2.37 | 2.46 | 2.34 | 2.36 | 2.21 | 2.32 | 2.34 | 2.37 |
| ENSG00000184984 | CHRM5 | 0.68 | 2.01 | 1.37 | 1.43 | 1.82 | 1.44 | 2.36 | 1.67 | 2.16 | 0.74 |
| ENSG00000171766 | GATM | 2.55 | 2.56 | 2.52 | 2.57 | 2.47 | 2.49 | 2.85 | 2.62 | 2.71 | 2.63 |
| ENSG00000117971 | CHRNB4 | 1.98 | 2.22 | 1.91 | 1.90 | 2.04 | 2.05 | 1.45 | 2.18 | 2.12 | 2.07 |
| ENSG00000090857 |  | 1.97 | 2.09 | 2.14 | 2.15 | 2.06 | 2.20 | 2.46 | 2.34 | 2.21 | 2.23 |
| ENSG00000181019 | NQO1 | 2.30 | 2.40 | 2.07 | 2.34 | 2.05 | 2.05 | 2.13 | 2.57 | 2.21 | 2.10 |
| ENSG00000170175 | CHRNB1 | 2.01 | 2.08 | 1.87 | 1.86 | 1.84 | 1.86 | 2.00 | 2.06 | 1.92 | 1.83 |
| ENSG00000108556 | CHRNE | 1.70 | 1.95 | 1.77 | 1.71 | 1.79 | 1.81 | 1.80 | 1.84 | 1.60 | 1.95 |
| ENSG00000133027 | PEMT | 2.11 | 2.24 | 2.04 | 2.14 | 1.96 | 2.14 | 2.06 | 2.10 | 2.07 | 2.17 |
| ENSG00000176974 | SHMT1 | 2.32 | 2.31 | 2.21 | 2.33 | 2.23 | 2.08 | 2.32 | 2.22 | 1.92 | 2.01 |
| ENSG00000129353 | SLC44A2 | 2.66 | 2.74 | 2.68 | 2.73 | 2.62 | 2.50 | 2.78 | 2.74 | 2.67 | 2.67 |
| ENSG00000130203 | APOE | 2.58 | 2.70 | 2.50 | 2.64 | 2.49 | 2.23 | 2.41 | 2.30 | 2.30 | 2.35 |
| ENSG00000130005 | GAMT | 1.90 | 1.93 | 1.94 | 1.97 | 1.60 | 2.02 | 2.10 | 1.91 | 1.94 | 1.87 |
| ENSG00000104879 | CKM | 2.12 | 2.00 | 1.33 | 2.07 | 1.95 | 1.84 | 2.02 | 1.89 | 1.81 | 1.85 |
| ENSG00000131069 | ACSS2 | 2.31 | 2.30 | 2.27 | 2.34 | 2.19 | 2.17 | 2.47 | 2.35 | 2.30 | 2.27 |
| ENSG00000101438 | SLC32A1 | 2.08 | 2.19 | 2.04 | 2.13 | 2.19 | 1.93 | 1.67 | 1.87 | 1.73 | 1.99 |
| ENSG00000154930 | ACSS1 | 2.40 | 2.49 | 2.31 | 2.45 | 2.36 | 2.34 | 2.67 | 2.45 | 2.56 | 2.61 |
| ENSG00000101204 | CHRNA4 | 1.99 | 1.86 | 1.88 | 1.99 | 1.80 | 1.72 | 1.76 | 1.80 | 1.81 | 1.82 |
| ENSG00000142192 | APP | 3.20 | 3.21 | 3.20 | 3.21 | 3.18 | 3.25 | 3.25 | 3.24 | 3.26 | 3.27 |
| ENSG00000205560 | CHKB | 2.10 | 2.03 | 2.10 | 2.11 | 2.08 | 2.14 | 2.27 | 2.09 | 2.16 | 2.11 |
| ENSG00000122121 | XPNPEP2 | 1.64 | 1.78 | 1.82 | 1.73 | 1.65 | 1.67 | 1.48 | 1.60 | 1.70 | 1.63 |
| ENSG00000102230 | PCYT1B | 2.29 | 2.31 | 2.29 | 2.35 | 2.04 | 2.30 | 2.42 | 2.45 | 2.33 | 2.32 |

| **Table S8.** mRNA expression of genes associated with metabolites not altered in schizophrenia | | | | | | | | | | |  | |
| --- | --- | --- | --- | --- | --- | --- | --- | --- | --- | --- | --- | --- |
|  |  |  |  |  |  |  |  |  |  |  | |  |
| Ensembl Gene ID | HGNC Symbol | Chimpanzee | | | | | Human | | | | | |
|  |  | C1 | C2 | C3 | C4 | C5 | H1 | H2 | H3 | H4 | | H5 |
| ENSG00000171793 | CTPS | 2.34 | 2.26 | 2.31 | 2.28 | 2.21 | 2.22 | 2.17 | 2.21 | 2.16 | | 2.15 |
| ENSG00000159423 | ALDH4A1 | 1.58 | 1.85 | 1.96 | 1.75 | 1.62 | 1.77 | 1.50 | 2.04 | 1.93 | | 1.83 |
| ENSG00000163873 | GRIK3 | 2.41 | 2.39 | 2.36 | 2.37 | 2.37 | 2.15 | 2.34 | 2.28 | 2.34 | | 2.36 |
| ENSG00000023909 | GCLM | 1.37 | 1.54 | 1.67 | 1.73 | 1.42 | 1.85 | 1.64 | 1.90 | 1.64 | | 1.44 |
| ENSG00000085491 | SLC25A24 | 1.62 | 1.85 | 1.60 | 1.84 | 1.76 | 1.86 | 1.87 | 1.83 | 1.87 | | 1.78 |
| ENSG00000143811 | PYCR2 | 2.09 | 2.16 | 2.05 | 2.10 | 1.95 | 2.01 | 2.08 | 1.93 | 2.01 | | 2.01 |
| ENSG00000143811 | PYCR2 | 1.69 | 1.65 | 1.45 | 1.72 | 1.52 | 1.46 | 1.62 | 1.67 | 1.51 | | 1.79 |
| ENSG00000084774 | CAD | 2.11 | 2.09 | 2.14 | 2.19 | 2.10 | 1.95 | 2.04 | 1.94 | 2.02 | | 1.88 |
| ENSG00000128683 | GAD1 | 2.73 | 2.71 | 2.65 | 2.61 | 2.78 | 2.71 | 2.45 | 2.68 | 2.72 | | 2.73 |
| ENSG00000128654 | MTX2 | 2.40 | 2.24 | 2.31 | 2.32 | 2.41 | 2.46 | 2.38 | 2.37 | 2.40 | | 2.41 |
| ENSG00000115419 | GLS | 2.95 | 2.95 | 2.92 | 2.90 | 2.97 | 3.03 | 2.94 | 3.05 | 3.03 | | 3.04 |
| ENSG00000021826 | CPS1 | 1.73 | 1.73 | 1.64 | 1.72 | 1.63 | 1.86 | 2.23 | 2.11 | 1.95 | | 1.90 |
| ENSG00000198380 | GFPT1 | 2.56 | 2.59 | 2.49 | 2.49 | 2.53 | 2.58 | 2.50 | 2.54 | 2.53 | | 2.55 |
| ENSG00000115840 | SLC25A12 | 2.66 | 2.66 | 2.58 | 2.60 | 2.70 | 2.67 | 2.62 | 2.67 | 2.70 | | 2.69 |
| ENSG00000197121 |  | 2.61 | 2.58 | 2.53 | 2.42 | 2.51 | 2.66 | 2.47 | 2.49 | 2.61 | | 2.63 |
| ENSG00000131389 | SLC6A6 | 2.11 | 2.31 | 2.32 | 2.35 | 2.41 | 2.29 | 2.43 | 2.07 | 2.20 | | 2.15 |
| ENSG00000188338 | SLC38A3 | 2.23 | 2.25 | 2.22 | 2.43 | 2.19 | 2.12 | 2.30 | 2.04 | 2.31 | | 2.23 |
| ENSG00000144746 | ARL6IP5 | 2.83 | 2.88 | 2.79 | 2.77 | 2.82 | 2.93 | 2.88 | 2.92 | 2.90 | | 2.92 |
| ENSG00000114120 | SLC25A36 | 2.58 | 2.53 | 2.50 | 2.55 | 2.67 | 2.53 | 2.47 | 2.47 | 2.54 | | 2.51 |
| ENSG00000163655 | GMPS | 2.73 | 2.74 | 2.63 | 2.69 | 2.75 | 2.73 | 2.68 | 2.71 | 2.69 | | 2.70 |
| ENSG00000152208 | GRID2 | 2.20 | 2.17 | 2.06 | 2.09 | 2.11 | 2.39 | 2.21 | 2.22 | 2.16 | | 2.22 |
| ENSG00000151729 | SLC25A4 | 2.79 | 2.83 | 2.74 | 2.71 | 2.87 | 2.80 | 2.67 | 2.74 | 2.78 | | 2.77 |
| ENSG00000163285 | GABRG1 | 2.48 | 2.61 | 2.30 | 2.46 | 2.62 | 2.20 | 2.52 | 2.67 | 2.71 | | 2.65 |
| ENSG00000151834 | GABRA2 | 2.72 | 2.72 | 2.73 | 2.66 | 2.83 | 2.76 | 2.71 | 2.76 | 2.86 | | 2.83 |
| ENSG00000109158 | GABRA4 | 2.77 | 2.75 | 2.72 | 2.65 | 2.78 | 2.85 | 2.61 | 2.75 | 2.79 | | 2.78 |
| ENSG00000128059 | PPAT | 2.32 | 2.17 | 2.20 | 2.15 | 2.42 | 2.21 | 2.16 | 2.11 | 2.21 | | 2.15 |
| ENSG00000079215 | SLC1A3 | 2.99 | 3.14 | 2.88 | 3.01 | 3.04 | 2.71 | 3.08 | 3.05 | 3.06 | | 3.04 |
| ENSG00000011083 | SLC6A7 | 2.15 | 2.18 | 2.28 | 2.06 | 1.95 | 2.35 | 2.07 | 2.19 | 2.23 | | 2.28 |
| ENSG00000145863 | GABRA6 | 1.79 | 1.98 | 1.68 | 1.79 | 1.71 | 1.33 | 1.63 | 1.77 | 1.80 | | 1.88 |
| ENSG00000022355 | GABRA1 | 2.93 | 2.93 | 2.87 | 2.90 | 2.92 | 3.02 | 2.89 | 2.94 | 3.02 | | 2.98 |
| ENSG00000113327 | GABRG2 | 3.00 | 3.02 | 2.99 | 2.95 | 3.03 | 3.06 | 2.95 | 3.07 | 3.06 | | 3.06 |
| ENSG00000152413 | HOMER1 | 2.85 | 2.78 | 2.74 | 2.72 | 2.92 | 2.84 | 2.67 | 2.70 | 2.73 | | 2.76 |
| ENSG00000129596 | CDO1 | 2.31 | 2.25 | 2.16 | 2.07 | 2.15 | 2.57 | 2.39 | 2.51 | 2.49 | | 2.47 |
| ENSG00000072682 | P4HA2 | 2.09 | 2.13 | 1.98 | 2.04 | 2.04 | 2.09 | 2.10 | 2.16 | 2.10 | | 2.22 |
| ENSG00000145864 | GABRB2 | 2.83 | 2.78 | 2.76 | 2.76 | 2.90 | 2.87 | 2.70 | 2.82 | 2.86 | | 2.83 |
| ENSG00000113262 | GRM6 | 1.90 | 1.96 | 2.00 | 1.95 | 1.82 | 1.91 | 1.71 | 1.94 | 1.86 | | 2.05 |
| ENSG00000131459 | GFPT2 | 2.15 | 2.19 | 2.18 | 2.19 | 2.31 | 2.31 | 2.26 | 2.15 | 2.16 | | 2.24 |
| ENSG00000112294 | ALDH5A1 | 2.51 | 2.60 | 2.46 | 2.57 | 2.45 | 2.60 | 2.53 | 2.56 | 2.58 | | 2.60 |
| ENSG00000196586 | MYO6 | 2.38 | 2.21 | 2.32 | 2.35 | 2.36 | 2.23 | 2.62 | 2.36 | 2.42 | | 2.29 |
| ENSG00000164418 | GRIK2 | 2.44 | 2.39 | 2.43 | 2.40 | 2.42 | 2.56 | 2.37 | 2.49 | 2.53 | | 2.49 |
| ENSG00000124493 | GRM4 | 2.08 | 2.22 | 2.13 | 2.00 | 2.12 | 2.10 | 1.87 | 2.10 | 2.00 | | 2.17 |
| ENSG00000001084 | GCLC | 2.42 | 2.43 | 2.34 | 2.44 | 2.37 | 2.29 | 2.53 | 2.36 | 2.39 | | 2.35 |
| ENSG00000146166 | GLULD1 | 1.91 | 1.92 | 1.81 | 1.85 | 1.82 | 1.60 | 1.62 | 1.81 | 1.69 | | 1.83 |
| ENSG00000146276 | GABRR1 | 1.85 | 1.96 | 1.65 | 1.60 | 1.80 | 1.75 | 1.66 | 0.79 | 0.93 | | 1.24 |
| ENSG00000111886 | GABRR2 | 1.12 | 1.41 | 1.46 | 1.47 | 1.46 | 1.63 | 1.66 | 1.70 | 1.71 | | 1.66 |
| ENSG00000185345 | PARK2 | 2.37 | 2.22 | 2.36 | 2.28 | 2.21 | 2.54 | 2.39 | 2.40 | 2.46 | | 2.45 |
| ENSG00000077800 | FKBP6 | 2.04 | 2.22 | 1.93 | 1.92 | 1.90 | 2.01 | 2.15 | 2.07 | 2.20 | | 2.16 |
| ENSG00000004864 | SLC25A13 | 2.41 | 2.35 | 2.32 | 2.39 | 2.15 | 2.05 | 2.45 | 2.08 | 2.28 | | 2.08 |
| ENSG00000070669 | ASNS | 3.01 | 2.95 | 2.93 | 2.95 | 2.98 | 3.05 | 2.93 | 2.97 | 3.02 | | 3.04 |
| ENSG00000137563 | GGH | 2.11 | 2.00 | 1.99 | 2.02 | 2.16 | 1.98 | 1.90 | 1.95 | 2.00 | | 1.92 |
| ENSG00000104524 | PYCRL | 2.01 | 2.02 | 2.03 | 1.92 | 1.89 | 1.97 | 1.82 | 1.80 | 1.96 | | 2.00 |
| ENSG00000106688 | SLC1A1 | 2.66 | 2.70 | 2.57 | 2.60 | 2.66 | 2.71 | 2.47 | 2.72 | 2.65 | | 2.68 |
| ENSG00000148053 | NTRK2 | 2.97 | 2.99 | 2.95 | 2.95 | 2.97 | 2.95 | 3.00 | 3.01 | 3.01 | | 3.03 |
| ENSG00000156052 | GNAQ | 3.02 | 3.06 | 3.03 | 3.00 | 3.00 | 3.03 | 2.99 | 3.07 | 3.06 | | 3.05 |
| ENSG00000148082 | SHC3 | 2.57 | 2.60 | 2.63 | 2.55 | 2.46 | 2.72 | 2.61 | 2.70 | 2.67 | | 2.69 |
| ENSG00000127080 | IPPK | 2.10 | 2.17 | 2.12 | 2.14 | 2.04 | 2.11 | 2.16 | 2.12 | 2.09 | | 2.12 |
| ENSG00000106789 | CORO2A | 2.23 | 2.06 | 2.19 | 2.16 | 2.13 | 1.89 | 1.98 | 2.10 | 2.03 | | 2.05 |
| ENSG00000136750 | GAD2 | 2.59 | 2.56 | 2.60 | 2.51 | 2.61 | 2.64 | 2.31 | 2.44 | 2.57 | | 2.57 |
| ENSG00000151151 | IPMK | 1.78 | 1.81 | 1.84 | 1.91 | 1.79 | 1.68 | 1.68 | 1.60 | 1.64 | | 1.71 |
| ENSG00000122884 | P4HA1 | 2.29 | 2.52 | 2.16 | 2.21 | 2.09 | 1.99 | 2.28 | 2.21 | 2.32 | | 2.34 |
| ENSG00000148672 | GLUDP5 | 3.06 | 3.08 | 3.06 | 3.06 | 3.09 | 3.01 | 3.13 | 3.12 | 3.14 | | 3.18 |
| ENSG00000148672 | GLUD1 | 3.06 | 3.08 | 3.06 | 3.06 | 3.09 | 3.01 | 3.13 | 3.12 | 3.14 | | 3.18 |
| ENSG00000059573 | ALDH18A1 | 2.20 | 2.30 | 2.17 | 2.21 | 2.20 | 2.25 | 2.17 | 2.32 | 2.27 | | 2.29 |
| ENSG00000152578 | GRIA4 | 2.48 | 2.45 | 2.50 | 2.41 | 2.52 | 2.66 | 2.43 | 2.48 | 2.52 | | 2.51 |
| ENSG00000149403 | GRIK4 | 2.19 | 2.19 | 2.25 | 2.24 | 1.94 | 2.14 | 2.18 | 2.14 | 2.13 | | 2.09 |
| ENSG00000177542 | SLC25A22 | 2.43 | 2.46 | 2.50 | 2.48 | 2.39 | 2.44 | 2.31 | 2.44 | 2.43 | | 2.52 |
| ENSG00000176697 | BDNF | 1.99 | 1.90 | 2.03 | 1.86 | 1.78 | 1.83 | 1.87 | 1.62 | 1.78 | | 1.90 |
| ENSG00000110436 | SLC1A2 | 3.17 | 3.21 | 3.15 | 3.18 | 3.24 | 2.96 | 3.20 | 3.22 | 3.22 | | 3.24 |
| ENSG00000175567 | UCP2 | 2.05 | 2.03 | 1.95 | 2.18 | 1.82 | 1.87 | 2.05 | 1.95 | 1.94 | | 1.75 |
| ENSG00000175564 | UCP3 | 2.01 | 1.93 | 1.94 | 1.65 | 1.89 | 1.95 | 1.86 | 1.76 | 1.89 | | 1.76 |
| ENSG00000168959 | GRM5 | 2.77 | 2.85 | 2.74 | 2.78 | 2.84 | 2.87 | 2.65 | 2.83 | 2.82 | | 2.85 |
| ENSG00000139344 | AMDHD1 | 1.84 | 1.95 | 1.89 | 1.69 | 1.84 | 1.40 | 1.85 | 1.48 | 1.77 | | 0.71 |
| ENSG00000179520 | SLC17A8 | 2.03 | 1.98 | 1.79 | 2.15 | 1.62 | 1.63 | 1.61 | 1.90 | 1.71 | | 2.11 |
| ENSG00000150086 | GRIN2B | 2.68 | 2.66 | 2.76 | 2.67 | 2.72 | 2.78 | 2.59 | 2.67 | 2.72 | | 2.74 |
| ENSG00000111371 | SLC38A1 | 2.92 | 2.83 | 2.91 | 2.89 | 2.94 | 2.93 | 2.91 | 2.91 | 2.96 | | 2.96 |
| ENSG00000135423 | GLS2 | 2.22 | 2.12 | 2.18 | 2.10 | 2.25 | 2.28 | 2.10 | 2.31 | 2.28 | | 2.29 |
| ENSG00000139874 | SSTR1 | 1.94 | 1.92 | 2.20 | 1.76 | 1.90 | 2.28 | 2.14 | 2.17 | 2.18 | | 2.14 |
| ENSG00000165555 | C14orf148 | 1.71 | 2.20 | 1.78 | 1.00 | 1.38 | 1.00 | 0.99 | 1.02 | 1.43 | | 1.92 |
| ENSG00000080824 | HSP90AA1 | 3.27 | 3.28 | 3.20 | 3.23 | 3.22 | 3.22 | 3.22 | 3.23 | 3.23 | | 3.22 |
| ENSG00000186297 | GABRA5 | 2.64 | 2.71 | 2.65 | 2.66 | 2.39 | 2.68 | 2.60 | 2.62 | 2.78 | | 2.76 |
| ENSG00000182256 | GABRG3 | 2.45 | 2.42 | 2.37 | 2.43 | 2.43 | 2.32 | 2.37 | 2.41 | 2.46 | | 2.53 |
| ENSG00000137825 | ITPKA | 2.06 | 2.06 | 2.04 | 1.97 | 2.09 | 2.34 | 1.98 | 2.05 | 2.06 | | 2.01 |
| ENSG00000103942 | HOMER2 | 2.20 | 2.24 | 2.26 | 2.24 | 2.37 | 2.30 | 2.24 | 2.27 | 2.21 | | 2.11 |
| ENSG00000183454 | GRIN2A | 2.75 | 2.73 | 2.77 | 2.72 | 2.84 | 2.77 | 2.67 | 2.73 | 2.72 | | 2.74 |
| ENSG00000178921 | PFAS | 2.13 | 2.26 | 2.16 | 2.27 | 2.17 | 2.16 | 2.10 | 2.20 | 2.10 | | 2.09 |
| ENSG00000131748 | STARD3 | 2.29 | 2.28 | 2.22 | 2.30 | 2.00 | 2.16 | 2.25 | 2.27 | 2.17 | | 2.06 |
| ENSG00000161653 | NAGS | 1.84 | 1.95 | 1.92 | 1.85 | 1.90 | 1.81 | 1.71 | 1.83 | 1.82 | | 1.86 |
| ENSG00000183048 | SLC25A10 | 2.29 | 2.14 | 2.35 | 2.47 | 1.85 | 1.87 | 1.98 | 1.85 | 1.91 | | 1.80 |
| ENSG00000161509 | GRIN2C | 2.00 | 2.15 | 2.19 | 2.13 | 2.01 | 1.78 | 2.30 | 1.98 | 2.10 | | 1.99 |
| ENSG00000185624 | P4HB | 2.27 | 2.44 | 2.17 | 2.30 | 2.23 | 2.47 | 2.46 | 2.57 | 2.56 | | 2.50 |
| ENSG00000123159 | GIPC1 | 2.39 | 2.40 | 2.45 | 2.48 | 2.34 | 2.36 | 2.36 | 2.37 | 2.36 | | 2.37 |
| ENSG00000105143 | SLC1A6 | 2.34 | 2.20 | 2.28 | 2.15 | 2.22 | 2.15 | 1.94 | 2.03 | 2.18 | | 2.14 |
| ENSG00000151151 | IPMK | 1.69 | 1.88 | 1.56 | 1.80 | 1.87 | 1.91 | 2.00 | 2.07 | 1.75 | | 1.82 |
| ENSG00000105655 |  | 1.92 | 1.89 | 2.09 | 1.90 | 1.79 | 2.24 | 1.85 | 1.97 | 1.97 | | 1.98 |
| ENSG00000051128 | HOMER3 | 2.28 | 2.17 | 2.17 | 2.34 | 2.15 | 1.83 | 2.22 | 1.79 | 1.98 | | 1.74 |
| ENSG00000161270 | NPHS1 | 1.53 | 1.69 | 1.55 | 1.67 | 1.41 | 1.56 | 1.38 | 1.64 | 1.57 | | 1.75 |
| ENSG00000161270 | PRODH2 | 1.53 | 1.69 | 1.55 | 1.67 | 1.41 | 1.56 | 1.38 | 1.64 | 1.57 | | 1.75 |
| ENSG00000171189 | GRIK1 | 2.18 | 2.12 | 2.08 | 2.16 | 2.31 | 2.31 | 2.04 | 2.19 | 2.34 | | 2.28 |
| ENSG00000100033 | PRODH | 2.03 | 1.77 | 1.93 | 1.98 | 2.01 | 1.60 | 2.11 | 1.98 | 1.87 | | 1.93 |
| ENSG00000100372 | SLC25A17 | 1.96 | 1.84 | 1.82 | 1.89 | 1.88 | 1.92 | 1.79 | 1.77 | 1.85 | | 1.73 |
| ENSG00000182890 | GLUD2 | 2.60 | 2.65 | 2.56 | 2.46 | 2.52 | 2.59 | 2.57 | 2.59 | 2.63 | | 2.62 |
| ENSG00000125675 | GRIA3 | 2.89 | 2.88 | 2.87 | 2.86 | 2.88 | 2.92 | 2.79 | 2.88 | 2.89 | | 2.93 |
| ENSG00000102078 | SLC25A14 | 2.09 | 2.07 | 1.96 | 1.84 | 2.01 | 2.08 | 1.94 | 2.05 | 2.18 | | 2.09 |
| ENSG00000047230 | CTPS2 | 2.36 | 2.49 | 2.45 | 2.46 | 2.42 | 2.39 | 2.47 | 2.37 | 2.32 | | 2.31 |
| ENSG00000102050 | PRAF2 | 2.20 | 2.18 | 2.20 | 2.23 | 2.06 | 2.23 | 2.21 | 2.21 | 2.12 | | 2.19 |
| ENSG00000102287 | GABRE | 1.97 | 1.76 | 1.19 | 1.65 | 1.67 | 1.57 | 1.67 | 1.31 | 0.97 | | 1.50 |
| ENSG00000176884 | GRIN1 | 2.58 | 2.65 | 2.58 | 2.57 | 2.50 | 2.67 | 2.62 | 2.57 | 2.64 | | 2.71 |
